# A novel *KCNT1* mutation in a Chinese family with severe autosomal-dominant nocturnal frontal lobe epilepsy

**DOI:** 10.1515/tnsci-2020-0182

**Published:** 2021-09-07

**Authors:** Na Xie, Weiwei Qin, Jianzhong Deng, Jinxing Qi, Dewang Niu, Guifeng Lu, Qun Wang

**Affiliations:** Department of Neurology, Anyang District Hospital of Puyang City, Henan, China; Department of Neurology, Henan Provincial People’s Hospital, Zhengzhou, Henan, China; Department of Neurology, Beijing Tiantan Hospital, Capital Medical University, Beijing, China

**Keywords:** KCNT1, severe ADNFLE, autosomal dominant, refractory epilepsy, psychiatric symptoms

## Abstract

We describe a Chinese family with severe autosomal-dominant nocturnal frontal lobe epilepsy (ADNFLE) and psychiatric problems in whom whole-exome family trio sequencing identified a heterozygous mutation in the potassium channel subfamily T, member 1 (*KCNT1*), a sodium-gated potassium channel gene, which was a novel missense mutation c.2153A>T (p. Asp718Val). The typical characteristics of the three patients in the family were refractory epilepsy, acquired cognitive impairment, and psychiatric problems, which include hallucinations and suicidal thoughts and behaviors. The age at onset was found to be earlier in son and daughter of the proband than that of the proband, as proven by the proband’s history of an epileptic seizure at the age of 16 years and her son’s and daughter’s history of seizures at the age of 8 years. Magnetic resonance imaging findings were negative for any abnormalities. Because of psychiatric symptoms, these three patients were administered risperidone at different times during their illness. The protestor’s son had tried fenofibrate treatment, but clinical remission was unclear. In summary, our findings broadened the mutation database in relation to *KCNT1* and implicated the sodium-gated potassium channel complex in ADNFLE, more broadly, in the pathogenesis of focal epilepsies.

## 1 Introduction

ADNFLE is a childhood-onset focal epilepsy syndrome characterized by clusters of motor seizures arising from sleep, usually occurring in individuals with normal intellect [1].

Mutations of the *KCNT1* have recently been identified in three families and a sporadic case with severe ADNFLE associated with intellectual and psychiatric problems [2].

In this report, we describe a family in which autosomal-dominant transmission of ADNFLE was explained by a novel mutation in *KCNT1*.

## 2 Case reports

**Figure 1:**
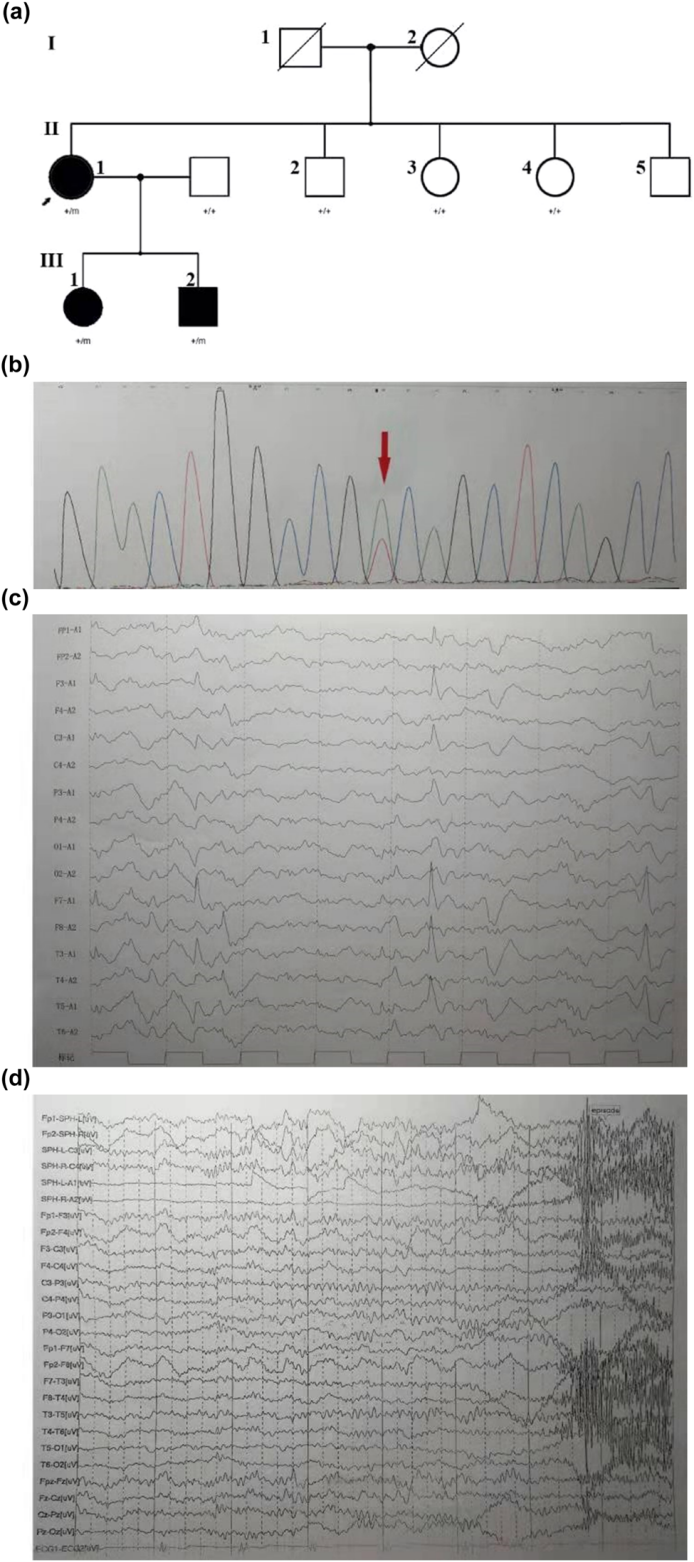
(a) Family pedigree in a novel mutation in KCNT1. The Chinese family includes three affected adults (II: 1, female, 47 years old; III: 1, female, 26 years old, III: 2, male, 17 years old). All subjects (II: 1, III: 1, III: 2) were tested by next generation sequencing. Comparison of the clinical features of the patients with missense mutation at c.2153A>T. (b) The DNA sequencing profile shows the c.2153A>T (p.Asp718Val) mutation in KCNT1. (c) The proband: interictal EEG shows double temporal apical slow waves. (d) The son: ictal EEG examined at 16-year-old. A short EEG seizure discharge begins at the right frontal region. The right anterior temporal, middle temporal, and right sphenoid spike rhythm lasted about 20 s, and then changed to full lead spike rhythm for about 40 s. Paroxysmal clinical manifestations: Sit up suddenly in sleep, lie down 20 s later, and gaze down with excessive movement of torso and proximal limbs, hands flexion and stiff, head left and right wobble, unconsciousness, last for about 48 s, and several similar episodes at night.

Proband: female, 47 years old, since the age of 16, suddenly felt suffocated and felt throat discomfort after falling asleep. She stared to the left, raised the left upper limb, sometimes moved both upper limbs, and straightened both lower limbs for a few seconds to 2 min. There were several attacks per day. Later episodes were frequent, sometimes manifested as turning the head to the left and staring to the left of the eyes, and these symptoms resolved in about 10 s. Sometimes the performance of the activity suddenly stopped or both hands twist spasm, both hands overstretch, left turn in circles. Each time lasted for tens of seconds. Sometimes, they present as tonic-clonic seizures in the extremities, lasting about a few minutes for relief, which was possibly induced and exacerbated by her menstruation. Prior to this, the patient did not receive any regular treatment, and her cognitive level was also found to be adequate for her work. In the past year, the frequency of seizures ranged from 2 to 3 times a day or 3 to 10 times a month to 4–5 times daily. Seizures evolved to frequent status epilepticus resulting in hospitalization. Then, she was given sodium valproate (0.4 g) three times a day; however, her epilepsy was not controlled. She was admitted to our hospital emergency department for frequent status epilepticus and psychiatric symptoms for 2 days. Mental motor development was normal. The patient was born in a healthy, non-inbred marriage with two healthy brothers and two healthy sisters. The obstetric history of the patient was normal, following an unremarkable pregnancy. Neuropsychological assessment revealed mild cognitive impairment [Montreal Cognitive Assessment (MoCA) 21/30] [3]. The scores of each parameter of the MoCA were as follows: visuospatial/executive (2/5), naming (2/3), attention (5/6), language (1/3), abstraction (1/2), delayed recall (5/5), and orientation (5/6). Other neurological examinations were normal and an MRI of the head showed no abnormality. The EEG showed normal background activity. High-amplitude spines and slow waves of 4 Hz occurred in the bilateral frontal regions in each sleep phase, sometimes affecting the anterior temporal and bilateral central regions (Figure 1c). Seizures are now better controlled after being on a regimen of carbamazepine 600 mg/day, valproate 1,500 mg/day, and risperidone 1 mg/day. Furthermore, risperidone was withdrawn when the psychiatric symptoms subsided.

Proband’s son, 17 years old: At the age of 8, there was a sudden feeling of breath-holding, throat failure, spitting during sleep without obvious inducement, and then panic, with double upper limbs dance-like movements, kicking, continue for 1–2 min to relieve. Every day or several days in a cluster attack, a dozen times to dozens of times each time. During the daytime, after strenuous activity or having a meal, had an attack, the head to one side askew (left and right sides have), consciousness was clear or fuzzy, stiff limbs shook, lasting about 5–6 min. At the age of 16, he developed mental symptoms, such as fear, locking the door back, cutting his wrists with scissors, and cutting his belly. His father was a healthy civil servant. Intelligence and neurological examination were normal. The ictal electroencephalogram (EEG) of the proband’s son was examined at 16 years of age (Figure 1d). The EEG showed normal background activity, moderate and high-amplitude sharp slow waves in the double frontal region, and the right anterior temporal lead during each sleep period. Several episodes were recorded in the monitoring, which was manifested as a sleep state, suddenly sitting up during the episode, lying down for 20 s, and staring with both eyes, accompanied by excessive movement of the trunk and proximal limbs, head shaking from side to side, and no response to calls, which lasted for about 40 s, synchronized with EEG, and fast wave rhythm was observed in the right midfrontal lead 5 s before the onset.

Proband’s daughter, 17 years old. When she was 8 years old, she often suddenly cried out, twisted her body, and sometimes suddenly felt sick or sat up for 1–2 min after falling asleep. They occurred in clusters at night. Sometimes, they were tonic-clonic seizures of the limbs. Seizures were more likely to occur during menstruation. The EEG background activity was normal, and the sleep EEG suggested a single spike in the forehead.

The proband’s son and daughter were admitted to the hospital emergency department for frequent status epilepticus and psychiatric symptoms several times. Her daughter had three suicide attempts, whereas her son had one when his mood fluctuated. Her daughter’s seizures were more controlled than before, but she still experienced an attack even after the combined use of sodium valproate 2,000 mg/day, carbamazepine 1,600 mg/day, and clonazepam 2 mg/day in the evening. In addition, her son’s seizures were also more controlled than before since the frequency and degree of the seizures were less than those in the past. He still experienced an attack even after the combined use of oxcarbazepine 1,800 mg/day, valproate 2,000 mg/day, levetiracetam 1,000 mg/day, and fenofibrate 0.1 g/day. Her daughter’s neurological examination upon admission revealed cognitive impairment and her neuropsychological assessment revealed mild cognitive impairment [Montreal Cognitive Assessment (MoCA) 19/30]. The MoCA scores were as follows: visuospatial/executive (4/5), naming (3/3), attention (5/6), language (3/3), abstraction (0/2), delayed recall (1/5), and orientation (3/6). On the other hand, her son’s neurological examination results were found to be unremarkable.

Blood DNA was extracted from the patient’s children to serve as the amplification template. Targeted sequence capture and next-generation sequencing technologies were used to sequence epilepsy-related genes. The results revealed a novel missense mutation in the *KCNT1* gene, which was a novel missense mutation c.2153A>T (p. Asp718Val). This mutation was found in the patient as well as her children (Figure 1a and b).

This variant was predicted by Sorting Intolerant From Tolerant (SIFT) and by MutationTaster to be damaging. The novel missense mutation c.2153A>T (p.Asp718Val) may disrupt protein function. This variant was predicted by MutationTaster, CADD score of 25.74, and SIFT score of 0.005 to be disease-causing. This mutation was not reported in HGMD Pro or PubMed. On the other hand, it was reported in either 1,000 genomes or the ExAC database.

**Ethical approval:** The research related to human use has been complied with all the relevant national regulations, institutional policies, and in accordance with the tenets of the Helsinki Declaration, and has been approved by the authors’ institutional review board or equivalent committee.**Informed consent:** Informed consent has been obtained from all individuals included in this study.

## 3 Discussion

Herein, we describe members of a Chinese family with severe autosomal-dominant nocturnal frontal lobe epilepsy (ADNFLE), who were identified with a novel KCNT1 gene mutation. The variant is not reported in ClinVar. The varian is of unknown significance in ACMG classification. However, the clinical manifestations, EEG, and genetic characteristics of this family were consistent with ADNFLE.

*KCNT1*, a gene that encodes a sodium-activated potassium channel that is highly expressed in the nervous system [4,5,6], is found in neurons of the frontal lobe [4]. Although it is not widely expressed in the cortex, it is consistent with its role in the pathogenesis of ADNFLE. It has also been thought to regulate hyperpolarization following repetitive firing. The C-terminal cytoplasmic domain of KCNT1 interacts with a protein network, including the Fragile X mental retardation protein, thus stimulating the KCNT1 channel [7]. *KCNT1* mutations were identified in three families and in a sporadic case with severe ADNFLE and psychiatric features in 2012. These mutations include the following: c.2782C>T (p.Arg928Cys) in an Australian British descent family, c.2386T>C (p.Tyr796His) in an Italian family, c.1193G>A (p.Arg398Gln) in the Israeli family, c.2688G>A (p.Met896Ile), and c.2688G>A (p.Met896Ile) in a sporadic case of severe sporadic nocturnal frontal lobe epilepsy (NFLE) and psychiatric problems [2]. In addition, KCNT1 mutations have also been reported in medial temporal lobe epilepsy. Hansen reported a novel phenotype with TLE, intellectual disability with early-adulthood onset being associated, and cerebellar ataxia in the KCNT1 channel. The mutation was heterozygous c.1694G>A mutation (p.Arg565His). The two patients in this report had hippocampal sclerosis and amygdala enlargement. Imaging lesions may be the structural cause of MTL epilepsy [8].

To date, heterozygous pathogenic variants of ADNFLE have been found in CHRNA4, CHRNB2, CHRNA2, KCNT1, DEPDC5, and CRH. Heron thinks that the age at onset of a seizure and the comorbidities associated with intellectual disability and psychiatric features in epileptic patients may be different between mutations affecting the nicotinic acetylcholine receptors (nAChRs) and KCNT1 [2]. Although intellectual disability and psychiatric disorders have been reported in ADNFLE [9,10], they are uncommon in families with ADNFLE that are associated with nAChR alterations. The phenotype associated with KCNT1 mutations differs from that associated with mutations affecting the nAChRs. Individuals carrying KCNT1 mutations have an earlier age at the onset of a seizure and frequently have significant comorbidities of intellectual disability and psychiatric features [2]. In contrast with the cases reported in the literature, our proband carrying KCNT1 mutation has a later age at onset of a seizure, which is 18 years of age. The clinical manifestations of this family member were refractory epilepsy and psychiatric symptoms. Genetic counseling is important for families carrying KCNT1 mutations.

Psychiatric comorbidity and cognitive impairment may occur, even if clinical neurologic examination and intelligence in ADNFLE are normal. The manifestations of the disease may vary greatly in the same family, and our patients had normal intelligence prior to the onset of seizures while some present with decreased intelligence with frequent seizures. It is still unknown whether the intellectual disability is the result of frequent seizures, gene mutations, or antiepileptic drugs. The onset of our proband was reduced at 48-year-old. This is consistent with the literature reports that ADNFLE is not progressive and that the attacks may become milder and less frequent as patients reach middle age.

ADNFLE is characterized by clusters of nocturnal motor seizures, varying from simple arousals from sleep to dramatic. The seizures of all members of the family were stereotyped and brief (several seconds to 5 min), which are often bizarre hyperkinetic events with dystonic or tonic features. The son and the proband may experience the aura of vomiting and laryngeal discomfort, respectively; the daughter and the proband possibly experienced an exacerbation of their menses.

ADNFLE is sensitive to the treatment of carbamazepine. However, carbamazepine alone does not control the symptoms. The proband’s son still had seizures after the combination of anti-epileptics. Fenofibrate, as an adjunctive therapy, reduced the seizure frequency in individuals with pharmaco-resistant ADNFLE/NFLE [11]. We tried to give fenofibrate to the proband’s son but clinical remission was unclear. In addition, Milligan reported that exposure to quinidine significantly reduces the gain of function for KCNT1 pathogenic variants implicated in ADNFLE and EIMFS [12]. Carbamazepine is the main therapeutic drug and it is often given at low doses; however, individuals with the CHRNA4 pathogenic variant p.Ser284Leu were found to be more responsive to zonisamide. Approximately 30% of individuals are resistant to AEDs. All patients in the family suffered from medically refractory epilepsy. The dosages of carbamazepine and oxcarbazepine were very high in all three patients, and the individuals required a trial of more than two appropriate AEDs. Addition of large amounts of anti-epileptic drugs did not address the seizures of our proband’s son leading us to switch to adjunctive fenofibrate therapy. After the addition of fenofibrate, the seizure attacks appeared to be more controlled than before, but clinical remission was unclear. Fenofibrate was discontinued after 3 months because the father thought it was a lipid-lowering drug. Owing to the short duration, the exact efficacy of fenofibrate in this patient could not be evaluated. All three patients had mental-related symptoms, and all of them were cured after short-term treatment with a small dose of risperidone. One year later, the protestor went to the doctor because of decreased interest and appetite. She was reexamined for hypothyroidism and has improved after receiving supplemental adenosin tablets. The protestor was clinical seizure-free. The protester’s son and daughter continued to have intermittent seizures. During the follow-up 2 years later, the protestor’s son had a suicide attempt and after being administered an adjusted antipsychotic medication, his condition improved. Reexamination of the protester’s son showed a slightly higher TSH level (8.03 µIU/mL) and normal TF3 and FT4 levels of thyroid function. The psychiatrist tried to add levothyroxine sodium tablets. At follow-up 2 years later, the protestor’s daughter had a slightly higher TSH level (7.25 µIU/mL) and normal TF3 and FT4 levels. Considering her low mood, we added a small dose of levothyroxine sodium tablets.

In conclusion, we identified a novel missense mutation in *KCNT1* in a Chinese family with ADNFLE. All members of this family had refractory epilepsy and severe mental symptoms. The thyroid function was normal at the beginning of the disease. However, it was not clear whether the different degrees of thyroid dysfunction in the follow-up were related to the disease. This finding provides additional evidence in understanding *KCNT1* pathogenesis better.
